# Psychometric properties of the World Health Organization quality of life assessment – brief in methadone patients: a validation study in northern Taiwan

**DOI:** 10.1186/1477-7517-10-37

**Published:** 2013-12-10

**Authors:** Tiffany Szu-Ting Fu, Yung-Change Tuan, Muh-Yong Yen, Wei-Hsin Wu, Chun-Wei Huang, Wei-Ti Chen, Chiang-Shan R Li, Tony Szu-Hsien Lee

**Affiliations:** 1 Department of Health Promotion and Health Education, National Taiwan Normal University, No. 162, He-ping East Road, Section 1, Taipei 10610, Taiwan; 2 Department of Psychiatry, New Taipei City Hospital, No.198, Yingshi Rd., Banqiao Dist., New Taipei City 220, Taiwan; 3 Department of Infection, Taipei City Hospital, Branch for Disease Control and Prevention, No 100 Kunming Street, Taipei 10844, Taiwan; 4 Department of Psychiatry, Ministry of Health and Welfare, Keelung Hospital, No 268 Sin 2nd Rd, Keelung City 20148, Taiwan; 5 Department of Psychiatry, Lotung Poh-Ai Hospital, 83 Nan Chang St., Lotung, Yilan 265, Taiwan; 6 School of Nursing, Yale University, New Haven, CT 06519, USA; 7 Department of Psychiatry, Yale University School of Medicine, 34 Park Street, New Haven 06519, USA

**Keywords:** Methadone, Psychometrics, Quality of life, WHOQOL-BREF, Injection drug users, Taiwan

## Abstract

**Background:**

Quality of life (QOL) is an important outcome measure in the treatment of heroin addiction. The Taiwan version of the World Health Organization Quality of Life assessment (WHOQOL-BREF [TW]) has been developed and studied in various groups, but not specifically in a population of injection drug users. The aim of this study was to analyze the psychometric properties of the WHOQOL-BREF (TW) in a sample of injection drug users undergoing methadone maintenance treatment.

**Methods:**

A total of 553 participants were interviewed and completed the instrument. Item-response distributions, internal consistency, corrected item-domain correlation, criterion-related validity, and construct validity through confirmatory factor analysis were evaluated.

**Results:**

The frequency distribution of the 4 domains of the WHOQOL-BREF (TW) showed no floor or ceiling effects. The instrument demonstrated adequate internal consistency (Cronbach’s alpha coefficients were higher than 0.7 across the 4 domains) and all items had acceptable correlation with the corresponding domain scores (*r* = 0.32-0.73). Correlations (*p* < 0.01) of the 4 domains with the 2 benchmark items assessing overall QOL and general health were supportive of criterion-related validity. Confirmatory factor analysis yielded marginal goodness-of-fit between the 4-domain model and the sample data.

**Conclusions:**

The hypothesized WHOQOL-BREF measurement model was appropriate for the injection drug users after some adjustments. Despite different patterns found in the confirmatory factor analysis, the findings overall suggest that the WHOQOL-BREF (TW) is a reliable and valid measure of QOL among injection drug users and can be utilized in future treatment outcome studies. The factor structure provided by the study also helps to understand the QOL characteristics of the injection drug users in Taiwan. However, more research is needed to examine its test-retest reliability and sensitivity to changes due to treatment.

## Background

Injecting use of heroin has been a risk factor for acquiring infectious diseases, including the human immunodeficiency virus (HIV) and hepatitis C virus (HCV) [1]. In Taiwan, it is estimated that 34% of the HIV cases are injection drug users (IDUs) [2]. To stem the spread of HIV, methadone maintenance treatment (MMT) was adopted in 2006 in Taiwan. Studies evaluating the efficacy and effectiveness of MMT have been centered on the objective outcome criteria, such as retention, death, heroin use, and crime rates [3]. However, it is not clear how IDU perceive the effects of treatment and, in particular, how treatments have impacted their quality of life [4]. Drug abuse or dependence is associated with multiple negative consequences physically and mentally as well as in social relations and work [5]. Although methadone alleviates the physical symptoms of opioid dependence [6], how it impacts the quality of life of IDUs remains to be evaluated. Therefore, it is important to consider subjective patients’ perception in the assessment of harm reduction programs by means of self-reported measures [7,8].

Because heroin addiction is a chronic and relapsing condition, which causes substantial impairments in both the physical and psychosocial health of the patients, the quality of life (QOL) scales have been used as an outcome measure to evaluate and to quantify the changes in patients’ functioning and well-being during treatment [9,10]. The generic QOL instruments commonly used in the MMT-related studies include the Nottingham Health Profile (e.g., [11,12]), the Quality of Well-Being Scale (e.g., [13]), the Medical Outcomes Study 36-Item Short-Form Health Survey (e.g., [14,15]), and the short version of the World Health Organization Quality of Life assessment (WHOQOL-BREF) (e.g., [16,17]). Of these generic QOL measures, only the WHOQOL is developed by first establishing the definition of subjective QOL as ‘an individual’s perception of their position in life in the context of the culture and value systems in which they live, and in relation to their goals, expectations, standards and concerns’ [18-20].

The WHOQOL contains 100 items (WHOQOL-100) [20]. To minimize the burden of the respondents and to ameliorate time constraint, a shortened, 26-item version, the WHOQOL-BREF, was developed [21]. The WHOQOL-BREF was translated for use in Taiwan (TW) according to the WHO international guidelines [22], and shown to have good test-retest reliability, internal consistency, content and discriminant validity [23]. The psychometric properties of the WHOQOL-BREF (TW) have been explored earlier (e.g., [24-26]). For example, Chen et al. [24] demonstrated adequate reliability and validity of the WHOQOL-BREF (TW) and that suggested that it may be used as a health indicator in different age groups from early adolescence to adulthood. In addition, it has been demonstrated that the instrument is suitable for the assessment of health status perceived by persons with spinal cord injury [27] and hemodialysis patients [28].

However, to our knowledge, few studies to date have provided evidence of the reliability and validity of the WHOQOL-BREF (TW) in IDUs enrolled in a MMT program. In order to use the instrument with the IDUs, the suitability of the WHOQOL-BREF (TW) needs to be validated prior to its clinical application to this population group [29]. The purpose of the present study was therefore to examine the item-response distribution, internal consistency, corrected item-domain correlation, criterion-related validity, and construct validity of the WHOQOL-BREF (TW) in IDUs under MMT.

## Methods

### Participants and procedure

A total of 599 eligible participants who fulfilled the Diagnostic and Statistical Manual of Mental Disorders (DSM) version IV criteria for heroin abuse from 4 methadone outpatient clinics in northern Taiwan were invited during the years 2008 and 2009 to participate in the study. Ethics approval for this study was obtained from the Institutional Review Board of Human Subjects Protection at the Taipei Medical University (P960205) and the Taipei City Hospital (TCHIRB-970404-E). 553 participants were interviewed and 46 withdrew their participation without signing the consent. All participants met the following eligibility criteria: heroin abuse/dependence, enrolled in methadone outpatient treatment, aged 18 or above and being able to read. They were enrolled in a MMT program at the time of interview. After completion, participants were reimbursed for $100 New Taiwan dollars (approximately 3 US dollars).

### Outcome measure

The self-administered WHOQOL-BREF (TW) (Additional file 1) contains 28 items, including 2 benchmark items assessing overall QOL and general health [22]. The remaining 26 items consist of 24 standard items from the original WHOQOL-BREF in 4 domains: physical health, psychological health, social relationships, and environment, plus 2 culturally relevant items: “Do you feel respected by others?” (social relationships domain), and “Are you usually able to get the things you like to eat?” (environment domain).

Scoring of the WHOQOL-BREF (TW) concerns “how good”, “how satisfied”, “how completely”, “how often” or “how much” the participant felt in the last 2 weeks and is rated on a 5-point Likert scale. The 2 benchmark items (overall QOL and general health) were calculated as a single score with a range of 1-5. Domain scores were calculated by multiplying the mean score for all items included in each domain by a factor of 4 [21]. Therefore, potential scores for each domain range from 4 to 20, with higher scores indicating better QOL.

### Data analysis

Descriptive analysis was used to report the demographic and clinical characteristics of the participants. The response distributions of domain and item scores were calculated to identify problematic items. Floor and ceiling effects were considered significant if the percentage of participants achieving the lowest and the highest scores exceeds 20% of the total sample [30]. To test reliability, the Cronbach’s alpha coefficient was used to evaluate the internal consistency of each domain of the WHOQOL-BREF (TW). Cronbach’s alpha values equal to or greater than 0.70 were considered satisfactory [31]. The corrected item-domain correlation was also reported to examine the homogeneity of the items in each domain. The criterion-related validity of the WHOQOL-BREF (TW) was evaluated by measuring the strength of Pearson’s *r* correlation between each item/domain scores and two criteria (i.e., overall QOL and general health). The strength of relationship of 0.40 or above was considered satisfactory (*r* ≥ 0.81-1.0 as excellent, 0.61-0.80 very good, 0.41-0.60 good, 0.21-0.40 fair and ≤ 0.20 poor) [31].

For construct validity, confirmatory factor analysis based on a maximum likelihood estimate, was conducted to validate whether the existing domain structure could be fit to the IDUs enrolled in a methadone treatment program while controlling for age, education, gender, HIV, HCV, duration of heroin use, methadone dose and days in MMT at study interview. The goodness-of-fit indicators include: the comparative fit index (CFI) and the non-normed fit index (NNFI) with values above 0.90 as well as the root mean square error of approximation (RMSEA) with values lower than 0.08 [32]. Model modifications were performed when the modification index suggests adding an error covariance between two items or adding a path from domain to item. Based on the two indices, the suggestion, with the greatest decrease in chi-square value, was selected to modify the model until the above criteria (CFI > 0.90, NNFI > 0.90, and RMSEA < 0.08) were met [33].

## Results

### Characteristics of the participants

Of participants, 510 completed the WHOQOL-BREF (TW) in a private room at the clinic of about 15 minutes and used in the analysis. The data of 43 patients were excluded from further analysis because they did not respond to all WHOQOL-BREF questions. Table 1 shows the demographic and clinical characteristics of these 510 participants. The mean age of the participants was 40.7 (standard deviation [SD] = 9.1) and mean duration of heroin use was 11.96 years (SD = 7.78). The average methadone dose was 51.62 mg/day (SD = 32.4) and mean duration of participating in the MMT was 183.86 days (SD = 150.90). Most were males (86.9%). The majority of the participants (72.7%) had completed at least 9 years of compulsory schooling; 12% were HIV positive and over 85% were HCV positive.

**Table 1:** Demographic and clinical characteristics of the participants (n = 510)

**Characteristics**	**Mean (S.D.)**
Age groups (year)	40.7 (9.1)
Duration of heroin use (years)	11.96 (7.78)
Average MMT dose (mg/day)	51.62 (32.40)
Duration in MMT (days)	183.86 (150.90)
	Number (%)
Gender	
Male	443 (86.9)
Female	67 (13.1)
Education	
Less than 9 years	133 (26.1)
At least 9 years	371 (72.7)
Missing replies	6 (1.2)
HCV	
Negative	31 (6.1)
Positive	435 (85.3)
Missing replies	44 (8.6)
HIV	
Negative	429 (84.1)
Positive	61 (12.0)
Missing replies	20 (3.9)

SD: standard deviation; HCV: hepatitis C virus; HIV: human immunodeficiency virus.

### Score distributions

As indicated in Table 2, the floor and ceiling effects in each domain score was low (0%-1.0%). For all WHOQOL-BREF (TW) items, the percentage of lowest and highest scores was, in general, below 20%. However, floor effects for the positive feelings facet (20.0%) and for the financial resources facet (21.2%) were observed. In addition, a ceiling effect for the dependence on medication or treatment facet (24.5%) was notable.

**Table 2:** Internal consistency and score distribution of the WHOQOL-BREF (TW) in IDU (n = 510)

**Domain and facet**	**α**	**Floor effect (%)**	**Ceiling effect (%)**
Overall quality of life		2.7	3.3
General health		4.1	2.0
Physical health domain	0.79	0.0	0.2
Pain and discomfort		2.7	13.5
Dependence on medication or treatment		2.9	24.5
Energy and fatigue		4.9	4.7
Mobility		4.3	9.4
Sleep and rest		7.5	3.5
Activities of daily living		1.8	3.5
Working capacity		3.1	5.1
Psychological health domain	0.78	0.0	0.0
Positive feelings		20.0	1.2
Spirituality, religion and personal beliefs		12.9	6.5
Thinking, learning, memory and concentration		6.7	4.3
Body image and appearance		3.7	12.5
Self-satisfaction		4.3	6.9
Negative feelings		6.5	3.9
Social relationships domain	0.76	0.8	1.0
Personal relationships		1.8	4.7
Sexual activity		5.9	4.1
Friend’s support		2.2	4.1
Respect^a^		6.3	2.7
Environment domain	0.87	0.0	0.2
Physical safety and security		9.0	2.9
Physical environments		8.0	4.3
Financial resources		21.2	3.5
Opportunities for new information and skills		5.7	3.5
Participation and support of leisure activities		6.1	3.3
Home environment		1.6	7.5
Heath and social care: availability and quality		1.4	5.1
Transportation		2.0	4.5
Eating^a^		1.0	14.3

WHOQOL-BREF (TW): the short Taiwan version of the World Health Organization Quality of Life assessment; IDU: injection drug user; ^a^Taiwanese national items.

### Reliability

The estimated values of Cronbach’s alpha for the physical health, psychological health, social relationships, and environment domains were 0.79, 0.78, 0.76, and 0.87 (Table 2), respectively, confirming adequate internal consistency of the instrument. On corrected item-domain correlation, all items had acceptable correlation coefficients (0.32-0.73) (data not shown in tables).

### Validity

The Pearson’s *r* correlation coefficients of the 4 WHOQOL-BREF (TW) domains and the criterion measures are shown in Table 3. All domain scores were fairly to moderately correlated with overall QOL (0.40 ≤ *r* ≤ 0.52, *p* < 0.01) and general health (0.41 ≤ *r* ≤ 0.57, *p* < 0.01). At the item level, all had fair to good relationships with the aforementioned indicators (0.20 ≤ *r* ≤ 0.50, *p* < 0.01) (data not shown in tables) except for the pain and discomfort facet with overall QOL (*r* = 0.11) (data not shown in tables) and for the dependence on medication or treatment facet with overall QOL (*r* = 0.12) (data not shown in tables).

**Table 3:** Criterion-related validity of the WHOQOL-BREF (TW) in IDU (n = 510)

**Domain**	**Criterion measure**	
	**Overall quality of life**	**General health**
Physical health domain	0.44	0.57
Psychological health domain	0.51	0.56
Social relationships domain	0.40	0.41
Environment domain	0.52	0.49

WHOQOL-BREF (TW): the short Taiwan version of the World Health Organization Quality of Life assessment; IDU: injection drug user. All correlations were significant at the 0.01 levels.

A summary of the selected goodness-of fit indices for confirmatory factor analysis is presented in Table 4. The results of the original model with CFI = 0.87, NNFI = 0.86, and RMSEA = 0.073, indicated that the existing domain structure with particular items did not adequately fit the IDUs. The model reached a good fit (CFI = 0.92, NNFI = 0.91, RMSEA = 0.06) when the physical safety and security facet (environment domain) was allowed to cross-load on the psychological health domain and when 5 pairs of error variances were allowed to covary (i.e., “pain and discomfort” and “dependence on medication or treatment”; “positive feelings” and “spirituality, religion and personal beliefs”; “physical safety and security” and “physical environments”; “energy and fatigue” and “physical environments”; “thinking, learning, memory and concentration” and “physical safety and security”). In the final proposed model, the four first-order factors were found to load highly and significantly on their second-order factors (standardized loadings for physical health, psychological health, social relationships, and environment domains were 0.93, 0.98, 0.92, and 0.88, *p* < 0.05, respectively) (data not shown in tables), suggesting adequate construct validity.

**Table 4:** Goodness-of-fit indices from confirmatory factor analysis

	**Best suggestion**	**χ**^**2**^	**RMSEA (90% CI)**	**CFI**	**NNFI**
Original model		1093.71	0.073 (0.068 ~ 0.078)	0.87	0.86
Modified model 1	add cov (Psyc7, En8)	1025.10	0.070 (0.065 ~ 0.075)	0.89	0.87
Modified model 2	add cov (En9, Phy10)	965.93	0.067 (0.062 ~ 0.072)	0.89	0.88
Modified model 3	add cov (Phy3, Phy4)	906.85	0.064 (0.060 ~ 0.069)	0.90	0.89
Modified model 4	add cov (Psyc5, Psyc6)	856.15	0.062 (0.057 ~ 0.067)	0.91	0.90
Modified model 5	add Psyc-En8	834.17	0.061 (0.056 ~ 0.066)	0.91	0.90
Final model	add cov (En8, En9)	812.49	0.060 (0.055 ~ 0.065)	0.92	0.91

RMSEA: root mean square error of approximation; CI: confidence interval; CFI: comparative fit index; NNFI: non-normed fit index.

Confirmatory factor analyses were performed while controlling for age, education, gender, HIV, HCV, duration of heroin use, methadone dose and days stayed in MMT at study interview.

## Discussion

The purpose of the current study is to examine the applicability of the WHOQOL-BREF (TW) in a group of IDUs enrolled in a MMT program. This is among the first reports of psychometric properties of the WHOQOL-BREF (TW) in this population group. Our findings suggest that the WHOQOL-BREF (TW) had acceptable reliability (internal consistency, and corrected item-domain correlation) and reasonable criterion-related validity, except for the item related to pain and discomfort and the item assessing dependence on medication or treatment. Although the original domain structure of the WHOQOL-BREF (TW) did not fit the data very well, there was a good fit after a few adjustments. Generally, the results support the reliability and validity of the WHOQOL-BREF (TW) for the IDU population and provide further information in regard to the IDU’s QOL.

There were no floor or ceiling effects for the 4 domains. However, the positive feelings and financial resources facets showed significant floor effects. Because hopelessness is one of the neuropsychiatric consequences of HCV/HIV infection or coinfection in drug users [34] and it has been found that most drug users have considerable financial strain [35-37], high values of the floor effect in the two items (i.e., “Do you enjoy your life? and “Do you have enough money for whatever you need?) were not unexpected. Similar to the finding in an earlier study, we found a notable ceiling effect in the dependence on the medication or treatment facet [24]. The high level of ceiling effect in the item (i.e., “Do you need medical treatment to cope with your daily life?”) was not too surprising given that the majority of our study population comprised a relatively larger number of middle-aged participants rather than the elderly.

Cronbach’s alpha coefficients for the 4 domains of the WHOQOL-BREF (TW) were greater than the recommended standard of 0.70, indicating satisfactory internal consistency of the instrument. This is consistent with a prior study conducted in Iran on 115 drug addicts, in which the alpha coefficients for 6 domains of WHOQoL-100 ranged from .78 to .84 [38]. All the items of the WHOQOL-BREF (TW) had item domain correlations of 0.3 or greater in our study, which were above the minimum value of 0.2 [39], suggesting that the item had reasonable convergence with the domain in which it is included. The reliability of the WHOQOL-BREF (TW) was confirmed, with the values comparable to prior results [24,25].

The validity of an instrument concerns whether a test measures what it is intended to [40]. In the evaluation of the criterion-related validity, the 4 domain scores of the WHOQOL-BREF (TW) showed statistically significant correlations with the chosen criteria (i.e., overall QOL and general health). The good associations between the physical health, psychological health, social relationships, environment domains, and the criterion measures confirm agreement but not redundancy of these QOL-specific domains. At the item level, however, 2 items (i.e., “To what extent do you feel that your pain prevents you in doing what you need to do?” and “How much do you need any medical treatment to cope with your daily life?”) showed weak criterion-related validity with overall QOL. Similar finding was also reported in a validation study of the Taiwanese audio player-assisted interview version of WHOQOL-BREF [41]. Because overall QOL involves a wide range of factors, it is possible that an individual item, which reflects only a narrow QOL facet, showed lower association with the criterion. In addition, chronic pain is prevalent among methadone patients [42] and is significantly associated with methadone daily dosage [43]. The weak relation between pain and discomfort with general health and overall QoL may reflect the low daily dose of methadone of our participants. Taken together, these findings lent support to the criterion-related validity of the WHOQOL-BREF (TW) for assessing QOL and general health in IDUs.

In regard to the construct validity of the WHOQOL-BREF (TW), our factor-analytic results indicated a marginal acceptable model fit [44], which are in line with the findings from several WHOQOL-BREF psychometric studies. For instance, in 908 Polish healthy and sick respondents aged 18-85, a CFI of 0.87 was reported by Jaracz et al. [45]. Skevington et al. [46] found identical CFI values of 0.863 and 0.864 in two random samples of over 5000 adults (mean age = 40) living in 23 countries. In exploring the factor structure of the Taiwanese version of the WHOQOL-BREF, Yao et al. [47] showed that the original factor structure needed revision (CFI = 0.886). In order to achieve a better model fit, it is necessary to add error correlations [48]. Through covarying the error variances for items from different domains and allowing two items to cross-load on other domains, Hwang et al. [25] showed an increase on the Bentler’s CFI from 0.85 to 0.90. A Dutch study of women with breast cancer [49] found that the revised model, with the additional covariances between error terms, better represented the sample data in comparison to the initial model.

To modify the fit indices of the original model, we added a cross-loading (physical safety and security on psychological) and 5 inter-correlations: “pain and discomfort” with “dependence on medication or treatment”, “positive feelings” with “spirituality, religion and personal beliefs”, “physical safety and security” with “physical environments”, “energy and fatigue” with “physical environments”, and “thinking, learning, memory and concentration” with “physical safety and security”. Although the association of the safety facet with the psychological health domain may suggest a deviation from the theoretical measurement structure, it informs us about the conceptualization of this item by the drug users. The correlated residuals with respect to two pairs of items suggest something in common to these items. However, the need to correlate errors to obtain a better-fitting model raises a question as to why items that overlap in content are necessary in the scale.

In addition to the 2 benchmark items, the short form of the WHOQOL-100 contains 24 items and each represents one facet of the original form. That is, the WHOQOL-BREF includes 24 different QOL dimensions and the factor structure of the WHOQOL-BREF is formed depending on the correlations among the 24 QOL facets. However, it is unlikely that the correlations between the 24 QOL dimensions are the same across studies because there are differences in the demographic or clinical characteristics of the participant groups. For example, if one considers a subgroup of younger people and a subgroup of patients with HIV infection, it is very likely the item “sexual activity” will be differently related to other 23 items. Although the associations between the 24 QOL dimensions might differ between participant groups due to various reasons, it does not undermine the validity of the scale, but further shows how the QOL facets may link together in a different way. With a substantial sample size for the confirmatory factor analysis [50], this study demonstrates that the WHOQOL-BREF (TW) is a suitable scale for IDUs even though a few modifications are needed.

The results of the current study need to be considered in light of some limitations. First, because the vast majority of participants were male drug users, it is not possible to analyze whether gender has an effect on the measurement of QOL in IDUs. The second limitation concerns the generalizability of the findings. The study participants were recruited from methadone outpatient clinics in one geographic area and this group of injection drug users who share not only similar drug use behaviors but also exposure to the same modality of treatment. Thus, the results may not be generalized to other samples recruited from a different region. Third, our reliability evaluation of the instrument was limited to the tests of internal consistency. Lastly, due to the lack of specific psychological and functional-related information about the participants, it is not clear if such factors could have impacted the results. With these considerations in mind, this study provides good support for using the WHOQOL-BREF (TW) in QOL assessment among drug users. More research needs to be carried out to further examine the remaining psychometric properties of the instrument, such as the test-retest reliability and sensitivity to changes due to MMT over time.

## Conclusions

This is among the first studies to evaluate psychometric characteristics of the WHOQOL-BREF (TW) in a population of drug users. Although a few items had floor and ceiling effects and there were slightly different patterns found in the confirmatory factor analysis, it appears that the WHOQOL-BREF (TW) is a reliable and valid instrument to measure QOL in IDUs. The factor structure provided by the study also helps us to understand the QOL characteristics of the drug users in Taiwan.

## Abbreviations

CFI: Comparative fit index; HCV: Hepatitis C virus; HIV: Human immunodeficiency virus; IDUs: Injection drug users; MMT: Methadone maintenance treatment; NNFI: Non-normed fit index; QOL: Quality of life; RMSEA: Root mean square error of approximation; SD: Standard deviation; TW: Taiwan; WHOQOL-BREF: The short version of the World Health Organization quality of life assessment.

## Competing interests

The authors declare that they have no competing interests.

## Authors’ contributions

TSTF carried out the literature searches of previous related work, undertook the statistical analysis, interpreted the findings, and wrote the manuscript. TYC, WHW, HCW, and MYY participated in the study design, data collection and analysis. WTC and CRL contribute to the conceptual framework and interpretation of the results. TSHL conceived the study, designed the research, draft and revised the manuscript. All authors read and approved the final manuscript.

## Supplementary Material

Additional file 1 An English translation of WHOQOL-BREF [Taiwan]. Click here for file
