# Supplementary material for: Psychometric properties of the World Health Organization quality of life assessment – brief in methadone patients: a validation study in northern Taiwan
Source: Harm Reduct J. 2013 Dec 10;10:37. doi: 10.1186/1477-7517-10-37 (PMC4029077; doi:10.1186/1477-7517-10-37)
Supplement: Additional file 1 — An English translation of WHOQOL-BREF [Taiwan]. [file 1477-7517-10-37-S1.pdf]

An English translation of WHOQOL-BREF [Taiwan]

1. How would you rate your quality of life?
   Very poor   Poor   Neither poor nor good   Good   Very Good
2. How satisfied are you with your health?
   Very satisfied   Dissatisfied   Neither satisfied nor dissatisfied   Satisfied   Very satisfied
3. To what extent do you feel that physical pain prevents you from doing what you need to do?
   Not at all   A little   A moderate amount   Very much   An extreme amount
4. How much do you need any medical treatment to function in your daily life?
   Not at all   A little   A moderate amount   Very much   An extreme amount
5. How much do you enjoy life?
   Not at all   A little   A moderate amount   Very much   An extreme amount
6. To what extent do you feel your life to be meaningful?
   Not at all   A little   A moderate amount   Very much   An extreme amount
7. Ho well are you able to concentrate?
   Not at all   Slightly   Moderately   Mostly   Completely
8. How safe do you feel in your daily life?
   Not at all   Slightly   Moderately   Mostly   Completely
9. How healthy is your physical environment?
   Not at all   Slightly   Moderately   Mostly   Completely
10. Do you have enough energy for everyday life?
   Not at all   Slightly   Moderately   Mostly   Completely
11. Are you able to accept your bodily appearance?
   Not at all   Slightly   Moderately   Mostly   Completely
12. Have you enough money to meet your needs?
   Not at all   Slightly   Moderately   Mostly   Completely
13. How available to you is the information that you need in our day-to-day life?
   Not at all   Slightly   Moderately   Mostly   Completely
14. To what extent do you have the opportunity for leisure activities?
   Not at all   Slightly   Moderately   Mostly   Completely
15. How well are you able to get around?
   Very poor   Poor   Neither poor nor good   Good   Very Good
16. How satisfied are you with your sleep?
   Very satisfied   Dissatisfied   Neither satisfied nor dissatisfied   Satisfied   Very satisfied
17. How satisfied are you with your ability to perform your daily living activities?
   Very satisfied   Dissatisfied   Neither satisfied nor dissatisfied   Satisfied   Very satisfied
18. How satisfied are you with your capacity for work?
   Very satisfied   Dissatisfied   Neither satisfied nor dissatisfied   Satisfied   Very satisfied

19. How satisfied are you with yourself?
    Very satisfied Dissatisfied Neither satisfied nor dissatisfied Satisfied Very satisfied
20. How satisfied are you with your personal relationships?
    Very satisfied Dissatisfied Neither satisfied nor dissatisfied Satisfied Very satisfied
21. How satisfied are you with your sex life?
    Very satisfied Dissatisfied Neither satisfied nor dissatisfied Satisfied Very satisfied
22. How satisfied are you with the support you get from your friends?
    Very satisfied Dissatisfied Neither satisfied nor dissatisfied Satisfied Very satisfied
23. How satisfied are you with the conditions of your living place?
    Very satisfied Dissatisfied Neither satisfied nor dissatisfied Satisfied Very satisfied
24. How satisfied are you with your access to health services?
    Very satisfied Dissatisfied Neither satisfied nor dissatisfied Satisfied Very satisfied
25. How satisfied are you with your transport?
    Very satisfied Dissatisfied Neither satisfied nor dissatisfied Satisfied Very satisfied
26. How often do you have negative feelings, such as blue mood, despair, anxiety, depression?
    Never Seldom Quite often Very Often Always
27. Do you feel respected by others?
    Not at all Slightly Moderately Mostly Completely
28. Are you usually able to get the things you like to eat?
    Never Seldom Quite often Very Often Always
